# Serum IgG subclass levels and risk of exacerbations and hospitalizations in patients with COPD

**DOI:** 10.1186/s12931-018-0733-z

**Published:** 2018-02-14

**Authors:** Fernando Sergio Leitao Filho, Seung Won Ra, Andre Mattman, Robert S. Schellenberg, Gerard J. Criner, Prescott G. Woodruff, Stephen C. Lazarus, Richard Albert, John E. Connett, Meilan K. Han, Fernando J. Martinez, Janice M. Leung, S. F. Paul Man, Shawn D. Aaron, Robert M. Reed, Don D. Sin, for the Canadian Respiratory Research Network (CRRN)

**Affiliations:** 1 Centre for Heart Lung Innovation, St. Paul’s Hospital, Vancouver, BC V6Z 1Y6 Canada; 2 Department of Medicine (Division of Respiratory Medicine), University of British Columbia, Vancouver, BC Canada; 3 Federal University of Ceará, Fortaleza, Ceará Brazil; 4 University of Ulsan College of Medicine, Ulsan University Hospital, Ulsan, South Korea; 5 Department of Pathology and Laboratory Medicine, University of British Columbia, Vancouver, BC Canada; 6 Department of Thoracic Medicine and Surgery, Lewis Katz school of Medicine at Temple University, Philadelphia, PA USA; 7 Department of Medicine, University of California San Francisco, San Francisco, CA USA; 8 Pulmonary Sciences and Critical Care Medicine, University of Colorado, Denver, CO USA; 9 School of Public Health, University of Minnesota, Minneapolis, MN USA; 10 Department of Internal Medicine, University of Michigan, Ann Arbor, MI USA; 11 Joan and Sanford I. Weill Department of Medicine, Weill Cornell Medical College, Cornell University, New York, NY USA; 12 Department of Medicine, University of Ottawa, Ottawa, ON Canada; 13 Division of Pulmonary and Critical Care Medicine, University of Maryland School of Medicine, Baltimore, MD USA

**Keywords:** IgG, IgG subclass deficiency, COPD, Exacerbation, Hospitalization

## Abstract

**Background:**

The literature is scarce regarding the prevalence and clinical impact of IgG subclass deficiency in COPD. We investigated the prevalence of IgG subclass deficiencies and their association with exacerbations and hospitalizations using subjects from two COPD cohorts.

**Methods:**

We measured IgG subclass levels using immunonephelometry in serum samples from participants enrolled in two previous COPD trials: Macrolide Azithromycin for Prevention of Exacerbations of COPD (MACRO; *n* = 976) and Simvastatin for the Prevention of Exacerbations in Moderate-to-Severe COPD (STATCOPE; *n* = 653). All samples were collected from clinically stable participants upon entry into both studies. IgG subclass deficiency was diagnosed when IgG subclass levels were below their respective lower limit of normal: IgG1 < 2.8 g/L; IgG2 < 1.15 g/L; IgG3 < 0.24 g/L; and IgG4 < 0.052 g/L. To investigate the impact of IgG subclass levels on time to first exacerbation or hospitalization, we log-transformed IgG levels and performed Cox regression models, with adjustments for confounders.

**Results:**

One or more IgG subclass deficiencies were found in 173 (17.7%) and 133 (20.4%) participants in MACRO and STATCOPE, respectively. Lower IgG1 or IgG2 levels resulted in increased risk of exacerbations with adjusted hazard ratios (HR) of 1.30 (95% CI, 1.10–1.54, *p* < 0.01) and 1.19 (95% CI, 1.05–1.35, *p* < 0.01), respectively in the MACRO study, with STATCOPE yielding similar results. Reduced IgG1 or IgG2 levels were also associated with increased risk of hospitalizations: the adjusted HR for IgG1 and IgG2 was 1.52 (95% CI: 1.15–2.02, *p* < 0.01) and 1.33 (95% CI, 1.08–1.64, *p* < 0.01), respectively for the MACRO study; in STATCOPE, only IgG2 was an independent predictor of hospitalization. In our multivariate Cox models, IgG3 and IgG4 levels did not result in significant associations for both outcomes in either MACRO or STATCOPE cohorts.

**Conclusions:**

Approximately 1 in 5 COPD patients had one or more IgG subclass deficiencies. Reduced IgG subclass levels were independent risk factors for both COPD exacerbations (IgG1 and IgG2) and hospitalizations (IgG2) in two COPD cohorts.

**Trial registration:**

This study used serum samples from participants of the MACRO (NCT00325897) and STATCOPE (NCT01061671) trials.

**Electronic supplementary material:**

The online version of this article (10.1186/s12931-018-0733-z) contains supplementary material, which is available to authorized users.

## Background

Chronic obstructive pulmonary disease (COPD) is one of the leading causes of morbidity and mortality worldwide, projected to become the third leading cause of death in 2020 [1]. The course of COPD is punctuated by episodes of acute exacerbations, which result in worse quality of life, accelerated disease progression, and increased risk of mortality [1, 2]. Hospitalizations due to severe exacerbations are associated with poor survival, with a 5-year mortality rate of 55% based on a previous study [3]. Respiratory tract infections are major triggers of COPD exacerbations [1, 4].

There is increasing evidence that immunoglobulins (Ig) play a crucial role in preventing infections, particularly sinopulmonary infections which likely relate to the continuous exposure of the anatomy involved to a vast range of pathogens [5–9]. Immunoglobulin G (IgG) is the predominant Ig class, corresponding to 75–80% of total Ig levels in blood, and consisting of four subclasses (IgG1, IgG2, IgG3, and IgG4) [10, 11]. The primary mechanisms by which IgG inactivates or eliminates pathogens involve activation of the complement system and opsonization of pathogens [12]. These processes result in target cell lysis and enhanced phagocytosis, respectively [10, 13].

Recently, we demonstrated that hypogammaglobulinemia (defined as total IgG levels < 7.0 g/L) was present in approximately 1 out of 4 patients with moderate to severe COPD [14]. More importantly, those patients with reduced IgG levels demonstrated 50% to 100% higher risk for both exacerbations and hospitalizations. The relationship between various subclasses of IgG and risk of exacerbations, however, remains unknown, as only a few articles with a number of limitations have described the frequency of IgG subclass deficiencies in COPD[15–17] and no prior studies have evaluated their impact on hospitalizations. Considering that COPD is a heterogeneous disease with several clinical phenotypes [18], we analyzed samples from patients who were at increased risk for exacerbations, and a second independent COPD cohort was also used to replicate our findings.

## Methods

### Study design

This is a retrospective study which used samples from participants of two COPD cohorts: Macrolide Azithromycin for Prevention of Exacerbations of COPD (MACRO – First cohort; ClinicalTrials.gov number: NCT00325897) [19] and Simvastatin for the Prevention of Exacerbations in Moderate-to-Severe COPD (STATCOPE – Replication cohort; ClinicalTrials.gov number: NCT01061671) [20]. Briefly, the MACRO trial tested azithromycin (250 mg daily), a macrolide antibiotic with immunomodulatory and antibacterial effects, whereas the STATCOPE trial investigated simvastatin (40 mg daily), compared to placebo for the prevention of COPD exacerbations. Both trials were double-blinded and placebo-controlled intervention studies. All participants had a diagnosis of COPD, aged 40 years or older, were current or former smokers (≥ 10 pack-year smoking history), and had a forced expiratory volume in 1 s (FEV_1_) < 80% of predicted values after bronchodilator use. Additionally, all subjects had to meet at least one of the following inclusion criteria: use of supplemental oxygen, previous treatment with systemic glucocorticoids or antibiotic agents for respiratory problems, or a history of an emergency department visit or hospitalization for COPD exacerbation.

### Exacerbation and hospitalization data

In both cohorts, the number and severity of exacerbations that occurred during follow-up were captured. The following scale was used to grade the severity of exacerbations: 1) mild – treatment conducted at home, with or without contacting a health care provider; 2) moderate – treatment requiring a visit to the emergency department; 3) severe – treatment requiring hospitalization; and 4) very severe – in which patients needed intensive care unit (ICU) admission. COPD-related hospitalizations were identified by capturing all events graded as severe and very severe exacerbations.

### IgG measurements

Baseline blood samples were collected from clinically stable participants upon entry into both studies. A total of 978 and 654 samples from the MACRO and STATCOPE cohorts, respectively, were available. Two samples from the MACRO trial (azithromycin arm) and one sample from the STATCOPE trial (placebo arm) were of insufficient quantity to allow measurements of all IgG subclasses and were thus not included in the analysis. In total, IgG levels were measured in 1629 samples, 976 from participants of MACRO (468 in the azithromycin arm; 508 in the placebo arm) and 653 from participants of STATCOPE (322 in the simvastatin arm; 331 in the placebo arm). Serum IgG subclass levels were measured using Binding Site IgG subclass (IgG1–4) reagents (Birmingham, UK) by immunonephelometry on a Siemens BNII nephelometer (BNII; Erlangen, Germany). All samples were processed in the clinical laboratory at St. Paul’s Hospital, Vancouver, British Columbia, Canada. The following normal ranges for IgG subclasses in adults were used: IgG1, 2.80–8.00 g/L; IgG2, 1.15–5.70 g/L; IgG3, 0.24–1.25 g/L; IgG4, 0.052–1.25 g/L [21]. These ranges were obtained from a healthy population and include mean levels ±2 standard deviations [22]. IgG subclass deficiency was diagnosed when IgG levels were below their respective lower limit of normal for each subclass: IgG1 < 2.80 g/L, IgG2 < 1.15 g/L, IgG3 < 0.24 g/L, and IgG4 < 0.052 g/L.

### Statistical analysis

IgG subclass levels were not normally distributed, and thus are presented as median and interquartile range. Categorical variables are shown as number and percentages. We used the Mann-Whitney U test and the Pearson′s Chi-square test to compare the IgG subclass levels and the frequency of IgG subclass deficiencies between the two cohorts, respectively. The Mann-Whitney U test was also applied to analyze the IgG subclass levels according to the exacerbation status (participants with zero or one exacerbation vs. participants with two or more exacerbations) and the occurrence of hospitalizations during follow-up. Additionally, we compared the rates of COPD exacerbations in person-years (expressed as the number of acute exacerbations divided by the duration of follow-up in years) between participants with IgG subclass levels below and above the lower limit of normal (LLN) using the Mann-Whitney U test.

To investigate the effect of IgG levels on time to first COPD exacerbation or hospitalization, we applied Cox proportional-hazards regression models to obtain a hazard ratio (HR) and its 95% confidence intervals (CI) associated with these outcomes for each IgG subclass. In our Cox models, we adjusted for covariates that were considered biologically relevant to the outcomes of interest (exacerbations and hospitalizations) or that presented with a *p* < 0.2 at the univariate analysis. The following variables were included in our multivariate models: IgG subclass levels (log-transformed), study drug (azithromycin in the derivation cohort, or simvastatin in the validation cohort), age, gender, ethnicity, smoking status, FEV_1_ (% of predicted value), long-term oxygen therapy, inhaled corticosteroid therapy at baseline, and history of systemic steroids in the year prior to enrollment. To investigate any possible effect of steroids (as described above) on IgG subclass levels, we also included an interaction term in our multivariate models. For IgG subclasses that demonstrated significant associations in our time to event analyses, we also obtained the adjusted predicted probabilities of COPD exacerbations or hospitalizations (using logistic regression models) and calculated the Pearson’s correlation coefficient between these probabilities and IgG-levels (log-transformed). SPSS version 22.0 for Windows (IBM Corp., Armonk, NY, USA) was used for data analysis. The level of statistical significance was set at *p* < 0.05 for all tests.

## Results

### Characteristics of participants and prevalence of IgG deficiency

The details of participant characteristics at baseline are presented in Table 1. In MACRO, the participants were older (65.5 ± 8.6 vs. 62.8 ± 8.4 years, *p* < 0.001), were more likely to be Caucasians (82.6% vs. 77.9%, *p* = 0.02) and had slightly lower FEV_1_ (39.8 ± 15.7% vs. 41.5 ± 17.8%, *p* = 0.049), and a higher rate of long-term domiciliary oxygen use (60.1% vs. 48.7%, *p* < 0.001) compared with participants enrolled in STATCOPE. Conversely, the prevalence of current smokers was higher in STATCOPE (28.8% vs. 21.2%, *p* < 0.001).

**Table 1:** Baseline characteristics of participants in the MACRO and STATCOPE cohorts

Variable	MACRO (*n* = 976)	STATCOPE (*n* = 653)	P*
Age, years	65.5 ± 8.6	62.8 ± 8.4	< 0.001
Gender (Male: Female) – no. (%)	584 (59.8):392 (40.2)	381 (58.3):272(41.7)	0.55
Ethnicity (Caucasian) – no. (%)	806 (82.6)	509 (77.9)	0.02
BMI, kg/m^2^	27.7 ± 6.2	27.1 ± 6.8	0.062
Current Smokers – no. (%)	207 (21.2)	188 (28.8)	< 0.001
FVC, % predicted	70.2 ± 18.1	71.1 ± 19.1	0.29
FEV_1_, % predicted	39.8 ± 15.7	41.5 ± 17.8	0.049
Long-term oxygen therapy – no. (%)	587 (60.1)	318 (48.7)	< 0.001
Systemic steroid use in previous year – no. (%)	822 (84.2)	550 (84.2)	0.99
Inhaled steroid use – no. (%)	764 (78.3)	484 (74.1)	0.06
IgG1 (g/L)	5.09 (2.45)	5.26 (2.70)	0.18
IgG2 (g/L)	2.57 (1.72)	2.61 (1.64)	0.82
IgG3 (g/L)	0.62 (0.49)	0.63 (0.51)	0.96
IgG4 (g/L)	0.21 (0.28)	0.22 (0.32)	0.99
Total IgG (g/L)	8.68 (3.72)	8.84 (4.00)	0.70

Values expressed as means ± SD. IgG levels expressed as median (interquartile range). *Student’s t-test, Mann–Whitney U-test (for comparisons of IgG levels), or Chi-square test, as appropriate. Definition of abbreviations: *BMI* body mass index, *FVC* forced vital capacity, *FEV*_1_ forced expiratory volume in one second, *IgG* immunoglobulin G

Total IgG and IgG subclass levels were similar between both cohorts (Table 2). Overall, 306 (18.8%) participants in the merged cohort (*n* = 1629) presented with at least one IgG subclass deficiency (173 (17.7%) and 133 (20.4%) in MACRO and STATCOPE, respectively)(Table 2). IgG3 deficiency was the most frequent, whereas IgG1 deficiency was the least common. We also identified cases showing two or more IgG subclasses deficiencies simultaneously. The majority of participants identified with an IgG subclass deficiency were also diagnosed with hypogammaglobulinemia (defined as total IgG < 7.0 g/L), especially participants presenting with IgG1 (72/74, *n* = 97.3%) or IgG2 (69/93, 74.2%) deficiencies. No differences regarding the frequency of IgG subclass deficiencies between both MACRO and STATCOPE cohorts were observed (Additional file 1: Table S1). The median and interquartile range for each IgG subclass according to the presence or absence of the related IgG subclass deficiency are shown in Additional file 2: Tables S2 (First and Replication cohorts) and Additional file 3: Table S3 (merged cohort).

**Table 2:** Frequency of IgG subclass deficiency according to the presence or absence of associated hypogammaglobulinemia in the merged cohort (MACRO and STATCOPE cohorts combined)

IgG abnormality	Merged Cohort (*n* = 1629)
IgG1 deficiency – no. (%)	74 (4.5%)
With hypogammaglobulinemia – no. (%)	72 (4.4%)
No hypogammaglobulinemia – no. (%)	2 (0.1%)
IgG2 deficiency – no. (%)	93 (5.7%)
With hypogammaglobulinemia – no. (%)	69 (4.2%)
No hypogammaglobulinemia – no. (%)	24 (1.5%)
IgG3 deficiency – no. (%)	124 (7.6%)
With hypogammaglobulinemia – no. (%)	72 (4.4%)
No hypogammaglobulinemia – no. (%)	52 (3.2%)
IgG4 deficiency – no. (%)	114 (7.0%)
With hypogammaglobulinemia – no. (%)	73 (4.5%)
No hypogammaglobulinemia – no. (%)	41 (2.5%)
One or more IgG subclass deficiency – no. (%)	306 (18.8%)
With hypogammaglobulinemia – no. (%)	197 (12.1%)
No hypogammaglobulinemia – no. (%)	109 (6.7%)
Two IgG subclass deficiencies combined – no. (%)	47 (2.9%)
With hypogammaglobulinemia – no. (%)	37 (2.3%)
No hypogammaglobulinemia – no. (%)	10 (0.6%)
Three IgG subclass deficiencies combined – no. (%)^a^	20 (1.2%)
All IgG subclass deficiencies combined – no. (%)^a^	4 (0.2%)

The normal range for IgG levels in adults used in this analysis were: IgG1, 2.8–8.0 g/L; IgG2, 1.15–5.70 g/L; IgG3, 0.24–1.25 g/L; IgG4, 0.052–1.250 g/L; total IgG, 7.0–16.0 g/L. ^a^All participants were diagnosed with hypogammaglobulinemia (total IgG < 7.0 g/L).

### IgG subclass levels according to exacerbation status and hospitalizations

In both cohorts, we detected significantly lower IgG1 and IgG2 levels among participants who developed two or more exacerbations during follow-up versus those who did not have any exacerbations or developed only one exacerbation (Additional file 4: Figure S1). Similar results were also observed in both cohorts according to hospitalization status, with participants who required hospitalizations during follow-up presenting with lower IgG1 and IgG2 subclass levels (Additional file 5: Figure S2).

**Fig. 1:**
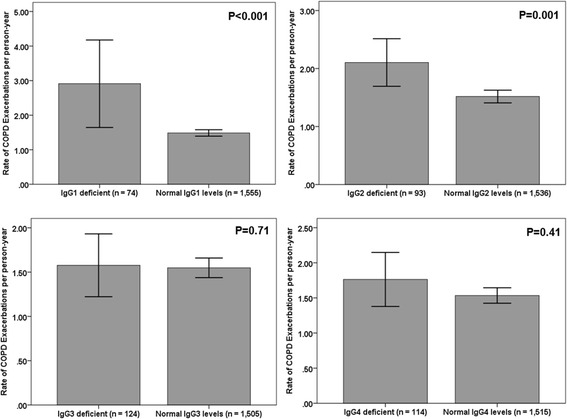
Comparison of rates of COPD exacerbations in person-years between participants with IgG subclass deficiency and normal IgG subclass levels in the merged dataset (MACRO and STATCOPE cohorts combined, *n* = 1629). Error bars represent 95% confidence interval

Given the low frequency of IgG subclass deficiency (< 10%) for all IgG subclasses in both cohorts, we merged both cohorts (*n* = 1629) and compared the rates of COPD exacerbations per person-year between participants according to the presence or not of IgG1–4 deficiencies. We observed significantly higher rates of exacerbations per person-year among participants with IgG1 deficiency (2.9 ± 5.46 vs. 1.48 ± 1.86, *p* < 0.001) and IgG2 deficiency (2.10 ± 1.99 vs. 1.51 ± 2.18, *p* = 0.001) compared to their counterparts with normal IgG1 and IgG2 levels (Fig. 1). Additionally, the frequency of IgG1 and IgG2 deficiency was also significantly higher among recurrent exacerbators compared to participants who remained stable or presented with only one exacerbation during the follow-up: IgG1–43/644 (6.7%) vs. 31/985 (3.1%), *p* = 0.001; IgG2–47/644 (7.3%) vs. 46/985 (4.7%), *p* = 0.025.

### Impact of IgG subclass levels on time to first COPD exacerbation and hospitalization

**Fig. 2:**
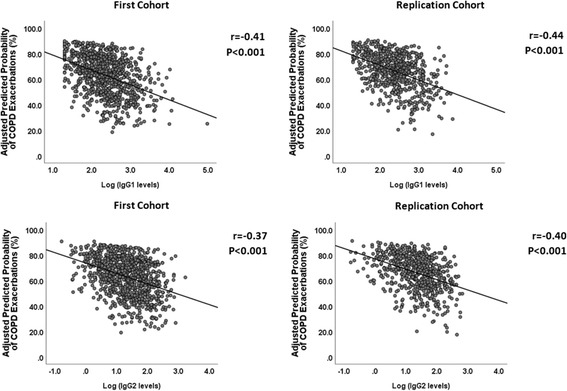
Correlation of IgG1 (upper panel) and IgG2 (lower panel) levels (log-transformed, base2) and the predicted risk of COPD exacerbations. For each participant, the predicted risks were calculated using logistic regression models with adjustments for the following covariates: study drug (azithromycin vs. placebo in MACRO - First cohort; simvastatin vs. placebo in STATCOPE - Replication cohort), age, gender, ethnicity (Caucasian vs. non-Caucasian), FEV_1_ (% of predicted values), smoking status (current vs. former smokers), oxygen dependence, inhaled corticosteroid use, and treatment with systemic steroids in previous year

**Fig. 3:**
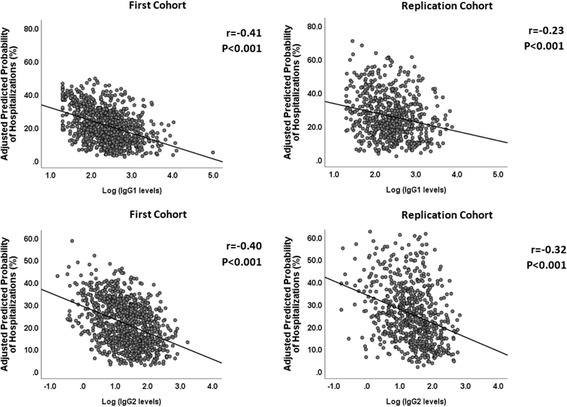
Correlation of IgG1 (upper panel) and IgG2 (lower panel) levels (log-transformed, base2) and the predicted risk of hospitalizations. For each participant, the predicted risks were calculated using logistic regression models with adjustments for the following covariates: study drug (azithromycin vs. placebo in MACRO - First cohort; simvastatin vs. placebo in STATCOPE - Replication cohort), age, gender, ethnicity (Caucasian vs. non-Caucasian), FEV_1_ (% of predicted values), smoking status (current vs. former smokers), oxygen dependence, inhaled corticosteroid use, and treatment with systemic steroids in previous year

The adjusted HRs for having a COPD exacerbation increased significantly with progressively lower IgG1 and IgG2 levels in MACRO (HR: 1.30, 95% CI: 1.10–1.54, *p* = 0.002; HR: 1.19, 95% CI: 1.05–1.35, *p* = 0.006, respectively) (Table 3). Since the IgG subclass levels in our models were expressed using a negative base-2 logarithmic scale, for every one-unit increase on this log scale, the IgG subclass levels decreased by 50% (negative log_2_2 = − 1). Thus, in MACRO, a 50% reduction of IgG1 levels was associated with a 30% higher adjusted HR for COPD exacerbations. A similar reduction in IgG2 levels resulted in a 19% higher adjusted HR for this outcome. These findings were replicated in STATCOPE, as the adjusted HRs related to COPD exacerbations were 1.25 (95% CI: 1.02–1.54, *p* = 0.035) and 1.17 (95% CI: 1.01–1.36, *p* = 0.046) for IgG1 and IgG2, respectively. IgG1 and IgG2 levels were also associated with hospitalizations in MACRO: the adjusted HR for every 50% reduction of IgG1 level was 1.52 (95% CI: 1.15–2.02, *p* = 0.004) and for IgG2 was 1.33 (95% CI: 1.08–1.64, *p* = 0.007) (Table 4). In STATCOPE, only decreased IgG2 levels resulted in a higher risk of hospitalizations (adjusted HR = 1.43, 95% CI: 1.12–1.83, p = 0.004). We also detected an inverse relationship between IgG levels (log-transformed) and the adjusted predicted risk of COPD exacerbations (Fig. 2) and hospitalizations (Fig. 3) in MACRO and STATCOPE cohorts (*p* < 0.05 for both outcomes).

**Table 3:** Unadjusted and adjusted hazard ratios for COPD exacerbations according to IgG subclass levels in the MACRO and STATCOPE cohorts

MACRO – Time to first Exacerbation				
IgG^a^	Univariate analysis		Multivariate analysis^b^	
	Hazard Ratio (95% CI)	*P*	Hazard Ratio (95% CI)	*P*
IgG1	1.43 (1.23–1.67)	< 0.001	1.30 (1.10–1.54)	0.002
IgG2	1.29 (1.15–1.45)	< 0.001	1.19 (1.05–1.35)	0.006
IgG3	0.99 (0.88–1.11)	0.84	0.95 (0.85–1.07)	0.39
IgG4	1.09 (1.02–1.17)	0.009	1.05 (0.98–1.13)	0.17
STATCOPE – Time to first Exacerbation				
IgG^a^	Hazard Ratio (95% CI)	*P*	Hazard Ratio (95% CI)	*P*
IgG1	1.45 (1.20–1.75)	< 0.001	1.25 (1.02–1.54)	0.035
IgG2	1.32 (1.15–1.52)	< 0.001	1.17 (1.01–1.36)	0.046
IgG3	1.10 (0.98–1.25)	0.11	0.99 (0.87–1.13)	0.90
IgG4	1.01 (0.94–1.08)	0.76	0.96 (0.89–1.04)	0.33

^a^IgG subclass levels expressed using a negative-log transformation (base 2), with one-unit increase on this log scale being equivalent to a 50% decrease of IgG subclass levels. ^b^Adjusted hazard ratios for IgG levels were calculated using a Cox Proportional Hazards model with adjustments for the following covariates: study drug (azithromycin vs. placebo in MACRO - First cohort; simvastatin vs. placebo in STATCOPE - Replication cohort), age, gender, ethnicity (Caucasian vs. Non-Caucasian), FEV_1_ (% of predicted values), smoking status (current vs. former smokers), oxygen dependence, inhaled corticosteroid use, and treatment with systemic steroids in previous year. Legend: 95% CI = 95% Confidence Interval

**Table 4:** Unadjusted and adjusted hazard ratios related to hospitalizations according to IgG subclass levels in the MACRO and STATCOPE cohorts

MACRO – Time to first Hospitalization				
IgG^a^	Univariate analysis		Multivariate analysis^b^	
	Hazard Ratio (95% CI)	*P*	Hazard Ratio (95% CI)	*P*
IgG1	1.51 (1.16–1.99)	0.003	1.52 (1.15–2.02)	0.004
IgG2	1.37 (1.12–1.67)	0.002	1.33 (1.08–1.64)	0.007
IgG3	1.03 (0.85–1.24)	0.79	1.01 (0.84–1.44)	0.90
IgG4	1.01 (0.89–1.12)	0.99	1.00 (0.88–1.12)	0.94
STATCOPE – Time to first Hospitalization				
IgG^a^	Univariate analysis		Multivariate analysis^b^	
	Hazard Ratio (95% CI)	*P*	Hazard Ratio (95% CI)	*P*
IgG1	1.33 (0.98–1.80)	0.07	1.27 (0.91–1.76)	0.16
IgG2	1.39 (1.11–1.74)	0.004	1.43 (1.12–1.83)	0.004
IgG3	0.97 (0.79–1.19)	0.75	0.90 (0.73–1.12)	0.36
IgG4	1.08 (0.95–1.22)	0.22	1.06 (0.93–1.20)	0.39

^a^IgG subclass levels expressed using a negative-log transformation (base 2), with one-unit increase on this log scale being equivalent to a 50% decrease of IgG subclass levels. ^b^Adjusted hazard ratios for IgG levels were calculated using a Cox Proportional Hazards model with adjustments for the following covariates: study drug (azithromycin vs. placebo in MACRO - First cohort; simvastatin vs. placebo in STATCOPE - Replication cohort), age, gender, ethnicity (Caucasian vs. Non-Caucasian), FEV_1_ (% of predicted values), smoking status (current vs. former smokers), oxygen dependence, inhaled corticosteroid use, and treatment with systemic steroids in previous year. Legend: 95% CI = 95% Confidence Interval

In our multivariate Cox models, IgG3 and IgG4 levels did not result in significant associations for both outcomes in either MACRO or STATCOPE cohorts. For these two IgG subclasses, we also calculated the HRs using both cohorts combined (*n* = 1629) and obtained similar HRs compared to analyzing the cohorts separately: IgG3 (exacerbations: HR = 0.97, 95% CI: 0.89–1.05, *p* = 0.53; hospitalizations: HR = 0.97, 95% CI: 0.84–1.12, *p* = 0.70); IgG4 (exacerbations: HR = 1.01, 95% CI: 0.96–1.06, *p* = 0.71; hospitalizations: 1.02, 95% CI: 0.94–1.12, *p* = 0.59).

We observed a trend for lower IgG1 subclass levels among participants who were treated with systemic steroids in the year prior to enrollment (MACRO - outcome: time to first exacerbation, *p* = 0.057; STATCOPE - outcome: time to first hospitalization, *p* = 0.07) (Additional file 6: Table S4). No statistically significant interactions were observed between IgG subclass levels and inhaled steroid therapy in both cohorts for either exacerbations or hospitalizations.

## Discussion

In this study, we found that IgG subclass deficiency was common in COPD, affecting approximately 1 in 5 patients who were at increased risk for COPD exacerbations. In addition, progressively lower IgG1 and IgG2 levels were associated with increased risk of exacerbations and hospitalizations in two different COPD cohorts. Moreover, the protective effect of IgG1 and IgG2 was even more pronounced on hospitalizations compared to exacerbations in both analyzed cohorts, emphasizing the potential role of IgG subclass levels in modulating the frequency and severity of COPD exacerbations.

Several prior reports have described the prevalence of IgG subclass deficiency in COPD patients [15–17]. O’Keeffe et al. observed an IgG subclass deficiency in 15 of 58 COPD patients (25.9%) [15]. Qvarfordt et al. measured IgG subclass levels in 33 smokers with chronic bronchitis and recurrent exacerbations, identifying an IgG subclass deficiency in 6 patients (18%) [16]. We obtained similar results, as at least one IgG subclass deficiency was detected in 17.7% and 20.4% of participants with COPD in MACRO and STATCOPE cohorts, respectively. Concerning the distribution of IgG subclass deficiency, we identified IgG3 as the most frequent (6.7% in MACRO and 9.0% of cases in STATCOPE), which has also been reported by other studies [16, 23]. Our findings suggest that the frequency of IgG abnormalities may be even higher among COPD patients who present with multiple exacerbations, as we detected a higher frequency of IgG1 and IgG2 deficiency among recurrent exacerbators. This is supported by a previous article, in which IgA, IgG, and IgM were measured in 42 COPD patients who had 2 or more moderate to severe exacerbations per year [24]. The authors detected in 29 patients (69%) evidence of an antibody deficiency syndrome, with 8 patients receiving a diagnosis of common variable immunodeficiency syndrome, whose diagnosis requires reduced total IgG levels [25]. Similarly, we observed significantly lower IgG1 and IgG2 levels among COPD participants who developed exacerbations and required hospitalizations in both cohorts.

In our Cox proportional hazards regression models, reductions in IgG1 and IgG2 levels in the First cohort were associated with an increased risk of COPD exacerbations and hospitalizations. These results remained statistically significant after adjustments for differences in age, gender, race, study drug, smoking status, oxygen dependence, FEV_1_ (% of predicted values), use of inhaled steroids and treatment with systemic steroids in the year prior to enrollment. Similar results were detected using a second independent cohort (thus increasing the validity of our findings) [26], with the exception that only IgG2 remained as an independent predictor of hospitalizations. Additionally, we also showed that participants with IgG1 and/or IgG2 deficiency presented with higher rates of COPD exacerbations per person-year during follow-up. To the best of our knowledge, this is the first report to demonstrate that lower IgG subclass levels are independent risk factors for both COPD exacerbations (IgG1 and IgG2) and hospitalizations (IgG2).

Our results are in line with some important concepts related to the biology of IgG subclasses. IgG1 and IgG2 are the major components of serum total IgG levels, explaining why patients with deficiencies in either one of these subclasses (or both) are more likely to present with reduced total IgG levels (i.e., hypogammaglobulinemia) [10, 11, 25]. We observed that 97.3% and 74.2% of patients diagnosed with IgG1 and IgG2 deficiencies (considering the merged cohort) also met criteria for hypogammaglobulinemia, respectively. Thus, patients with lower IgG1 and IgG2 levels, through a reduction in total IgG levels, may present with an increased risk of COPD exacerbations and hospitalizations, as these outcomes are mostly triggered by respiratory infections [4]. This is consistent with a previous work of our group, in which COPD patients with hypogammaglobulinemia showed a higher frequency of COPD exacerbations and hospitalizations compared to patients with normal total IgG levels [14]. Additionally, patients with IgG2 deficiency usually present with impaired polysaccharide responses, leading to a higher susceptibility to infections caused by encapsulated pathogens (e.g., *Streptococcus pneumoniae* and *Haemophilus influenzae* type B) [10, 12, 13], which are frequently implicated in bacterial COPD exacerbations [4].

It has been shown that not all patients with lower IgG subclasses present with recurrent infections, with some healthy individuals also being diagnosed with one or more IgG subclasses deficiencies [11]. Conversely, there is a rationale for evaluating patients with recurrent infections for possible IgG subclass deficiency. Kim JH et al. observed that one-third of patients with chronic airway diseases (asthma, COPD, or asthma COPD overlap syndrome) who had received a previous diagnosis of IgG subclass deficiency (*n* = 59) presented with a history of recurrent respiratory infections [23]. Additionally, these patients showed a higher frequency of hospitalizations and a faster FEV_1_ decline during follow-up. Our results indicate that IgG1 or IgG2 subclass deficiency may contribute to a higher exacerbation risk, and thus we advocate that measurement of IgG subclass levels be considered in any COPD patient with a history of several exacerbations or previous hospitalizations. Moreover, in this scenario, even if a COPD patient shows normal total IgG levels, it is still possible that an IgG1 or IgG2 subclass deficiency may be present, potentially contributing to an increased propensity to COPD exacerbations. In a previous article, Vendrell et al. found that 11% of patients with bronchiectasis of unknown etiology with normal total IgG levels (*n* = 107) still had an antibody production deficiency, with 50% of those presenting with IgG2 deficiency [27].

The management of IgG subclass deficiency includes vaccinations, prophylactic antibiotics, treatment of allergy and sinopulmonary disease (if present) and cautious use of immunoglobulin G replacement therapy - IVIG (for patients who persist with recurrent infections despite these interventions) [25]. In one previous study, administration of IVIG reduced the risk of respiratory infections by over 50% in 92/132 adults with IgG subclass deficiency presenting with recurrent infections [28]. In another study, IVIG significantly decreased the frequency of moderate to severe exacerbations by nearly 90% in 14 COPD patients (from an annual COPD exacerbation rate of 4.7 ± 3.1 to 0.6 ± 1.0 per patient)[29]. Interestingly, 64.3% of the participants (9/14) had evidence of hypogammaglobulinemia.

Our study had several limitations. Firstly, we were only able to measure IgG subclass levels at baseline, and thus we cannot determine how IgG subclass levels fluctuate over the course of COPD or with the implementation of various COPD treatments. Secondly, we only measured IgG subclass levels once, despite standard laboratory recommendations that two measurements be performed at least 1 month apart to confirm deficiency [11, 25]. Thirdly, we did not investigate whether patients with IgG subclass deficiency had impaired humoral immunity, usually evaluated by measuring antibody levels after a pneumococcal vaccination [11, 13, 25]. Finally, since this is a retrospective, observational study, we can only describe an association between low serum IgG1 or IgG2 levels and COPD exacerbations. Although IgG subclass deficiency may serve as a biomarker to identify subjects at increased risk of exacerbation, our study was not designed to determine if low serum IgG subclass levels are linked on a causal pathway to COPD exacerbations. It is worth mentioning we analyzed samples from moderate-to-severe COPD patients who were at increased risk of COPD exacerbations; thus the effect of lower IgG levels on the outcomes of COPD patients with mild disease or without a history of prior exacerbations was not evaluated by our study.

## Conclusions

Our data support that IgG subclass deficiency is relatively common among COPD patients and that decreased IgG1 and IgG2 levels result in an increased risk of adverse outcomes in COPD. Future prospective studies are needed to better elucidate the impact of these IgG subclass deficiencies on the management of high-risk COPD patients and whether they represent a modifiable risk factor for COPD exacerbations and hospitalizations through IVIG.

## Additional files

Additional file 1: Table S1. Prevalence of IgG subclass deficiencies in MACRO and STATCOPE cohorts. (DOCX 15 kb)

Additional file 2: Table S2. Median and interquartile range related to each IgG subclass according to the presence or absence of corresponding IgG subclass deficiency in MACRO and STATCOPE cohorts. (DOCX 15 kb)

Additional file 3: Table S3. Median and interquartile range related to each IgG subclass according to the presence or absence of corresponding IgG subclass deficiency in the merged dataset (MACRO and STATCOPE cohorts combined). (DOCX 15 kb)

Additional file 4: Figure S1. Comparison of IgG subclass levels according to exacerbation status in MACRO – First cohort (left panel) and STATCOPE – Replication cohort (right panel). Error bars represent 95% confidence interval. (DOCX 205 kb)

Additional file 5: Figure S2. Comparison of IgG subclass levels according to hospitalization status in MACRO – First cohort (left panel) and STATCOPE – Replication cohort (right panel) cohorts. Error bars represent 95% confidence interval. (DOCX 243 kb)

Additional file 6: Table S4. Interactions between IgG subclass levels and inhaled steroid use at enrollment and use of systemic steroids in previous 12 months for both outcomes of interest (time to first exacerbation and time to first hospitalization). (DOCX 15 kb)

## Abbreviations

CI: Confidence intervals
COPD: Chronic obstructive pulmonary disease
FEV_1_: Forced expiratory volume in 1 s
HR: Hazard ratio
Ig: Immunoglobulins
IgG: Immunoglobulin G
IVIG: Immunoglobulin G replacement therapy
LLN: Lower limit of normal
MACRO: Macrolide Azithromycin for Prevention of Exacerbations of COPD trial
STATCOPE: Simvastatin for the Prevention of Exacerbations in Moderate-to-Severe COPD trial
