# Supplementary material for: Serum IgG subclass levels and risk of exacerbations and hospitalizations in patients with COPD
Source: Respir Res. 2018 Feb 14;19:30. doi: 10.1186/s12931-018-0733-z (PMC5813358; doi:10.1186/s12931-018-0733-z)
Supplement: Supplementary file 1 — Prevalence of IgG subclass deficiencies in MACRO and STATCOPE cohorts. (DOCX 15 kb) [file 12931_2018_733_MOESM1_ESM.docx]

**Table S1. Prevalence of IgG subclass deficiencies in MACRO and STATCOPE cohorts**

| IgG abnormality | MACRO (n=976)  First Cohort (n=976) | STATCOPE (n=653)  Replication Cohort | P |
| --- | --- | --- | --- |
| IgG1 deficiency – no. (%) | 44 (4.5) | 30 (4.6) | 0.93 |
| IgG2 deficiency – no. (%) | 52 (5.3) | 41 (6.3) | 0.42 |
| IgG3 deficiency – no. (%) | 65 (6.7) | 59 (9.0) | 0.08 |
| IgG4 deficiency – no. (%) | 64 (6.6) | 50 (7.7) | 0.39 |
| One or more IgG subclass deficiency – no. (%) | 173 (17.7) | 133 (20.4) | 0.18 |
| Two or more IgG subclass deficiencies combined – no. (%) | 38 (3.9) | 33 (5.0) | 0.26 |
| Two IgG subclass deficiencies combined – no. (%) | 26 (2.7) | 21 (3.2) | N/A |
| Three IgG subclass deficiencies combined – no. (%) | 10 (1.0) | 10 (1.5) | N/A |
| All IgG subclass deficiencies combined – no. (%) | 2 (0.2) | 2 (0.3) | N/A |

The normal range for IgG subclasses in adults used in this analysis were: IgG1, 2.8 - 8.0 g/L; IgG2, 1.15 - 5.70 g/L; IgG3, 0.24 - 1.25 g/L; IgG4, 0.052 - 1.250 g/L. Legend: N/A = not applicable.
