# Supplementary material for: Serum IgG subclass levels and risk of exacerbations and hospitalizations in patients with COPD
Source: Respir Res. 2018 Feb 14;19:30. doi: 10.1186/s12931-018-0733-z (PMC5813358; doi:10.1186/s12931-018-0733-z)
Supplement: Supplementary file 2 — Median and interquartile range related to each IgG subclass according to the presence or absence of corresponding IgG subclass deficiency in MACRO and STATCOPE cohorts. (DOCX 15 kb) [file 12931_2018_733_MOESM2_ESM.docx]

**Table S2 – Median and interquartile range related to each IgG subclass according to the presence or absence of corresponding IgG subclass deficiency in MACRO and STATCOPE cohorts**

|  | MACRO | STATCOPE |
| --- | --- | --- |
| IgG1  IgG1 deficient (< 2.80 g/L)  Normal IgG1 levels (≥ 2.8 g/L) |  |  |
|  | 2.49 (0.19) | 2.61 (0.27) |
|  | 5.19 (2.15) | 5.37 (2.62) |
| IgG2  IgG2 deficient (< 1.15 g/L)  Normal IgG2 levels (> 1.15 g/L) |  |  |
|  | 0.97 (0.24) | 0.99 (0.27) |
|  | 2.63 (1.69) | 2.70 (1.50) |
| IgG3  IgG3 deficient (< 0.24 g/L)  Normal IgG3 levels (≥ 0.24 g/L) |  |  |
|  | 0.19 (0.05) | 0.20 (0.06) |
|  | 0.66 (0.46) | 0.69 (0.45) |
| IgG4  IgG4 deficient (< 0.052 g/L)  Normal IgG4 levels (≥ 0.052 g/L) |  |  |
|  | 0.038 (0.007) | 0.038 (0.001) |
|  | 0.230 (0.295) | 0.238 (0.322) |
