# Supplementary material for: Serum IgG subclass levels and risk of exacerbations and hospitalizations in patients with COPD
Source: Respir Res. 2018 Feb 14;19:30. doi: 10.1186/s12931-018-0733-z (PMC5813358; doi:10.1186/s12931-018-0733-z)
Supplement: Supplementary file 3 — Median and interquartile range related to each IgG subclass according to the presence or absence of corresponding IgG subclass deficiency in the merged dataset (MACRO and STATCOPE cohorts combined). (DOCX 15 kb) [file 12931_2018_733_MOESM3_ESM.docx]

**Table S3 – Median and interquartile range related to each IgG subclass according to the presence or absence of corresponding IgG subclass deficiency in the merged dataset (MACRO and STATCOPE cohorts combined)**

|  | Merged Dataset |
| --- | --- |
| IgG1  IgG1 deficient (< 2.80 g/L, n=74)  Normal IgG1 levels (≥ 2.8 g/L, n=1,555) |  |
|  | 2.52 (0.23) |
|  | 5.26 (2.51) |
| IgG2  IgG2 deficient (< 1.15 g/L, n=93)  Normal IgG2 levels (≥ 1.15 g/L, n=1,536) |  |
|  | 0.98 (0.25) |
|  | 2.67 (1.60) |
| IgG3  IgG3 deficient (< 0.24 g/L, n=124)  Normal IgG3 levels (≥ 0.24 g/L, n=1,505) |  |
|  | 0.19 (0.06) |
|  | 0.67 (0.45) |
| IgG4  IgG4 deficient (< 0.052 g/L, n=114)  Normal IgG4 levels (≥ 0.052 g/L, n=1,515) |  |
|  | 0.038 (0.004) |
|  | 0.234 (0.307) |
