# Supplementary material for: Serum IgG subclass levels and risk of exacerbations and hospitalizations in patients with COPD
Source: Respir Res. 2018 Feb 14;19:30. doi: 10.1186/s12931-018-0733-z (PMC5813358; doi:10.1186/s12931-018-0733-z)
Supplement: Supplementary file 4 — Comparison of IgG subclass levels according to exacerbation status in MACRO – First cohort (left panel) and STATCOPE – Replication cohort (right panel). Error bars represent 95% confidence interval. (DOCX 205 kb) [file 12931_2018_733_MOESM4_ESM.docx]

**Figure S1. Comparison of IgG subclass levels according to exacerbation status in MACRO – First cohort (left panel) and STATCOPE – Replication cohort (right panel). Error bars represent 95% confidence interval.**

**P=0.005**


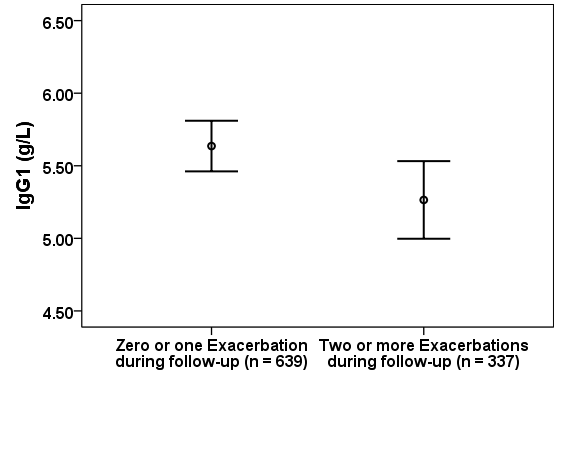

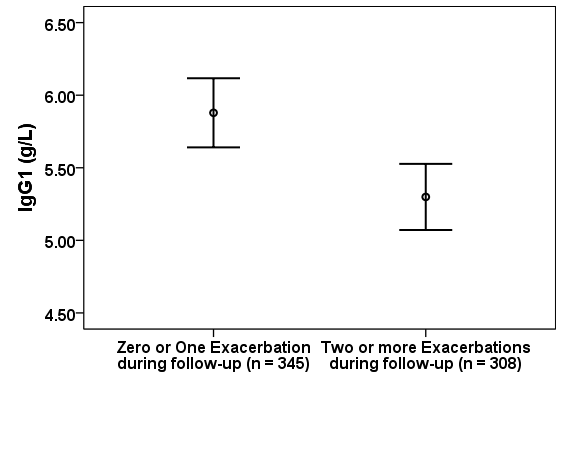

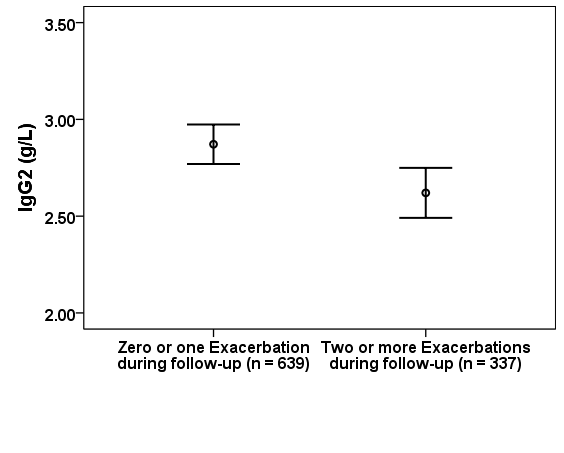

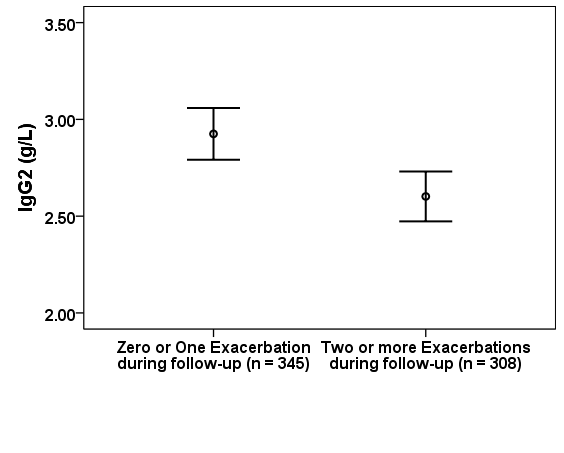


**P=0.001**

**P=0.001**

**P<0.001**

**P=0.005**


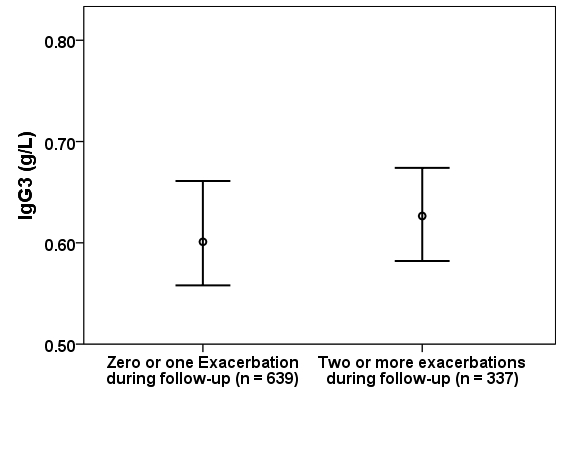

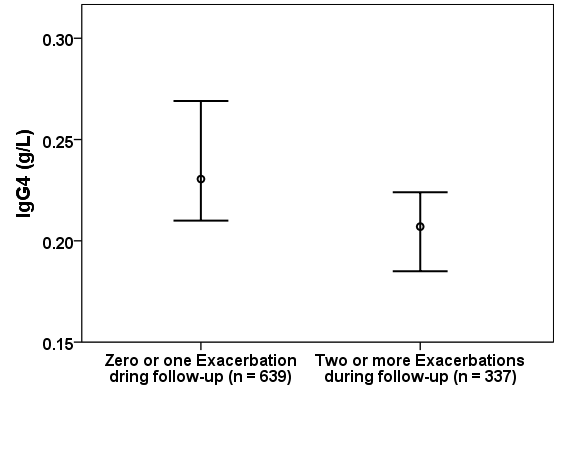


**P=0.15**

**P=0.19**

**P=0.28**


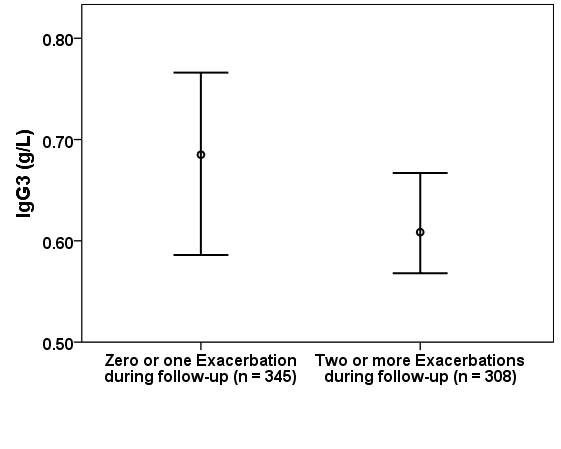


**P=0.12**


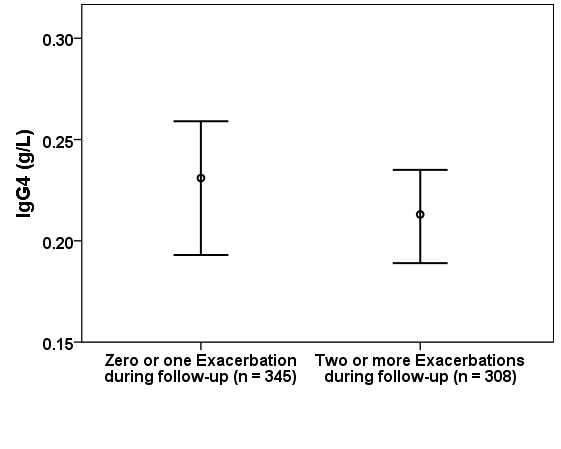


**P=0.28**
