# Supplementary material for: Serum IgG subclass levels and risk of exacerbations and hospitalizations in patients with COPD
Source: Respir Res. 2018 Feb 14;19:30. doi: 10.1186/s12931-018-0733-z (PMC5813358; doi:10.1186/s12931-018-0733-z)
Supplement: Supplementary file 5 — Comparison of IgG subclass levels according to hospitalization status in MACRO – First cohort (left panel) and STATCOPE – Replication cohort (right panel) cohorts. Error bars represent 95% confidence interval. (DOCX 243 kb) [file 12931_2018_733_MOESM5_ESM.docx]

**Figure S2. Comparison of IgG subclass levels according to hospitalization status in MACRO – First cohort (left panel) and STATCOPE – Replication cohort (right panel) cohorts. Error bars represent 95% confidence interval.**


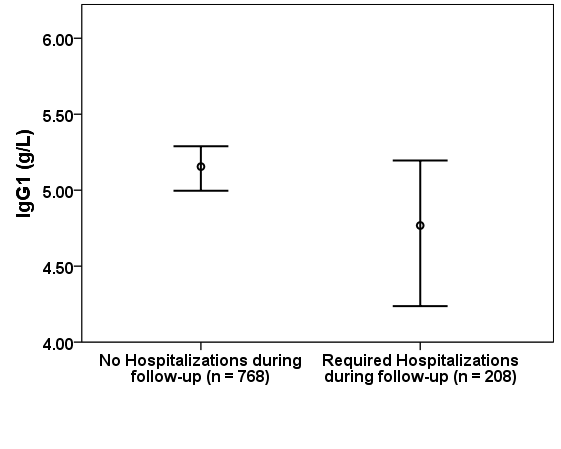

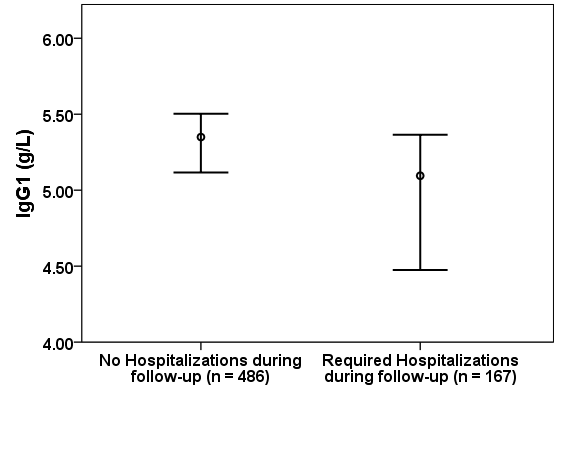

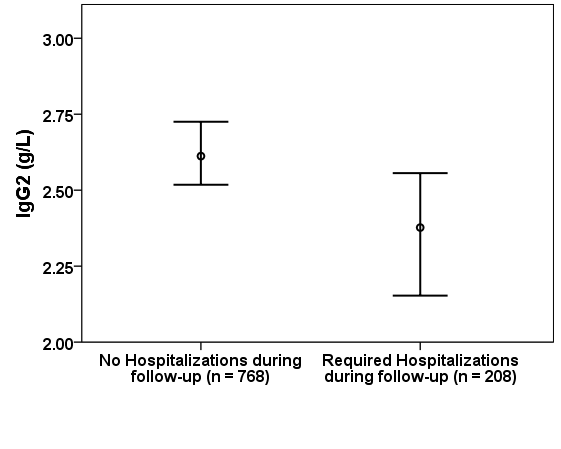

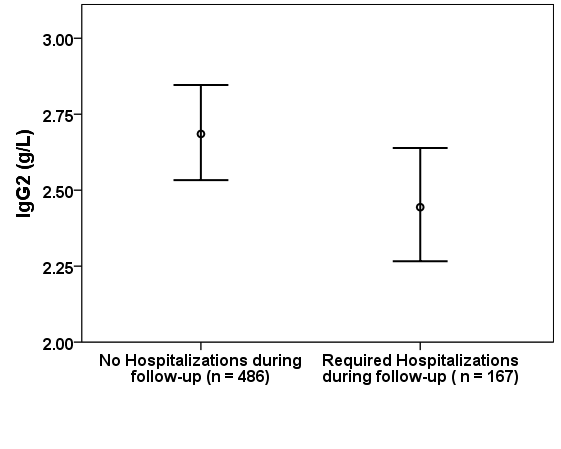

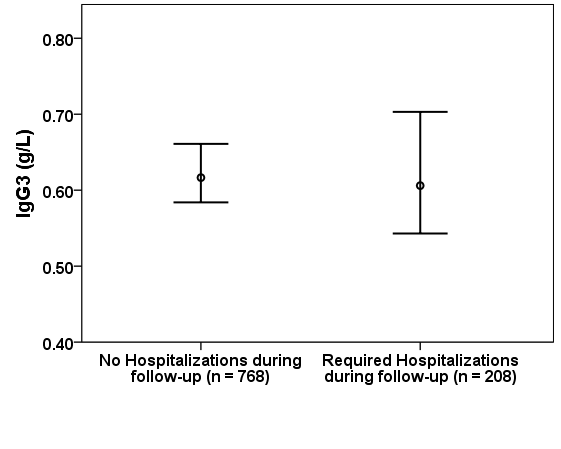

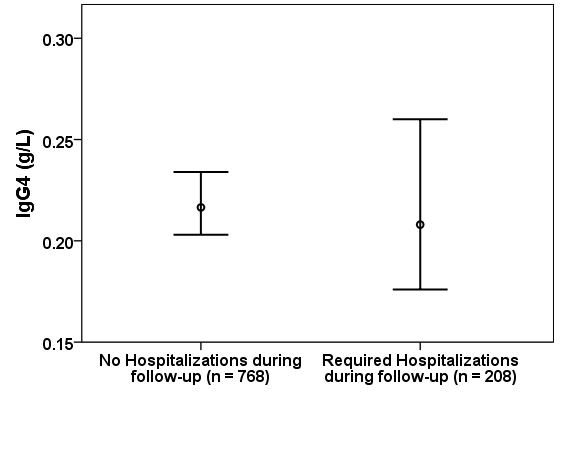

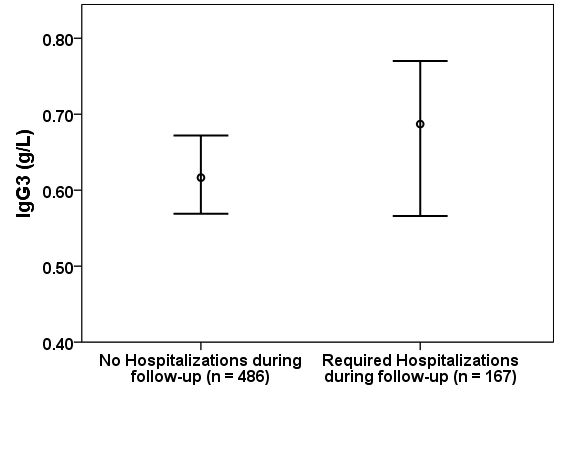

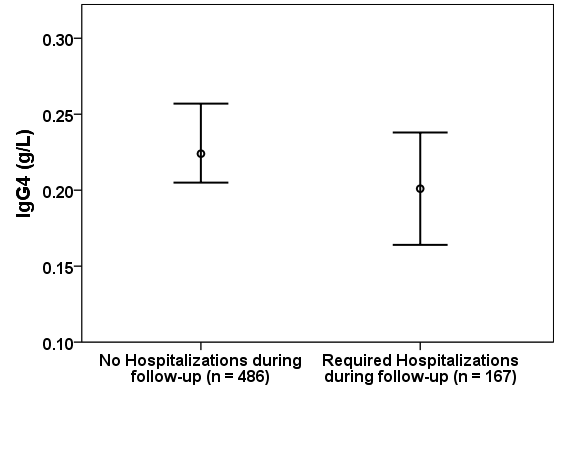


**P=0.002**

**P=0.002**

**P=0.86**

**P=0.99**

**P=0.04**

**P=0.009**

**P=0.19**

**P=0.52**
