# Supplementary material for: Serum IgG subclass levels and risk of exacerbations and hospitalizations in patients with COPD
Source: Respir Res. 2018 Feb 14;19:30. doi: 10.1186/s12931-018-0733-z (PMC5813358; doi:10.1186/s12931-018-0733-z)
Supplement: Supplementary file 6 — Interactions between IgG subclass levels and inhaled steroid use at enrollment and use of systemic steroids in previous 12 months for both outcomes of interest (time to first exacerbation and time to first hospitalization). (DOCX 15 kb) [file 12931_2018_733_MOESM6_ESM.docx]

**Table S4 – Interactions between IgG subclass levels and inhaled steroid use at enrollment and use of systemic steroids in previous 12 months for both outcomes of interest (time to first exacerbation and time to first hospitalization)**

| Interactions | MACRO (n=976)  First Cohort | STATCOPE (n=653)  Replication Cohort |
| --- | --- | --- |
| **Outcome: Time to first Exacerbation** |  |  |
| IgG1 * Inhaled steroid use at enrollment | P=0.17 | P=0.21 |
| IgG1 * Systemic steroid use in previous 12 months | P=0.057* | P=0.28 |
| IgG2 * Inhaled steroid use at enrollment | P=0.62 | P=0.10 |
| IgG2 * Systemic steroid use in previous 12 months | P=0.27 | P=0.50 |
| IgG3 * Inhaled steroid use at enrollment | P=0.82 | P=0.20 |
| IgG3 * Systemic steroid use in previous 12 months | P=0.99 | P=0.52 |
| IgG4 * Inhaled steroid use at enrollment | P=0.77 | P=0.32 |
| IgG4 * Systemic steroid use in previous 12 months | P=0.74 | P=0.76 |
| **Outcome: Time to first Hospitalization** |  |  |
| IgG1 * Inhaled steroid use at enrollment | P=0.64 | P=0.26 |
| IgG1 * Systemic steroid use in previous 12 months | P=0.15 | P=0.07** |
| IgG2 * Inhaled steroid use at enrollment | P=0.28 | P=0.77 |
| IgG2 * Systemic steroid use in previous 12 months | P=0.66 | P=0.65 |
| IgG3 * Inhaled steroid use at enrollment | P=0.47 | P=0.64 |
| IgG3 * Systemic steroid use in previous 12 months | P=0.77 | P=0.84 |
| IgG4 * Inhaled steroid use at enrollment | P=0.50 | P=0.49 |
| IgG4 * Systemic steroid use in previous 12 months | P=0.30 | P=0.46 |

*Treatment with systemic steroids in previous 12 months resulted in a trend for lower IgG1 levels (Cohort: MACRO; outcome: time to first exacerbation; Cohort: STATCOPE; outcome: time to first hospitalization).
